# A candidate chromosome inversion in Arctic charr (*Salvelinus alpinus*) identified by population genetic analysis techniques

**DOI:** 10.1093/g3journal/jkab267

**Published:** 2021-07-28

**Authors:** Matthew C Hale, Matthew A Campbell, Garrett J McKinney

**Affiliations:** 1 Department of Biology, Texas Christian University, Fort Worth, TX 76129, USA; 2 Department of Animal Science, University of California Davis, Davis, CA 95616, USA; 3 University of Alaska Museum, University of Alaska Fairbanks, Fairbanks, AK 99775, USA; 4 National Research Council Research Associateship Program, Northwest Fisheries Science Center, National Marine Fisheries Service, National Oceanic and Atmospheric Administration, Seattle, WA 98112, USA

**Keywords:** chromosomal inversion, salmonids, population genomics, RAD-seq, structural variants

## Abstract

The “genomics era” has allowed questions to be asked about genome organization and genome architecture of non-model species at a rate not previously seen. Analyses of these genome-wide datasets have documented many examples of novel structural variants (SVs) such as chromosomal inversions, copy number variants, and chromosomal translocations, many of which have been linked to adaptation. The salmonids are a taxonomic group with abundant genome-wide datasets due to their importance in aquaculture and fisheries. However, the number of documented SVs in salmonids is surprisingly low and is most likely due to removing loci in high linkage disequilibrium when analyzing structure and gene flow. Here we re-analyze RAD-seq data from several populations of Arctic charr (*Salvelinus alpinus*) and document a novel ∼1.2 MB SV at the distal end of LG12. This variant contains 15 protein-coding genes connected to a wide-range of functions including cell adhesion and signal transduction. Interestingly, we studied the frequency of this polymorphism in four disjointed populations of charr—one each from Nunavut, Newfoundland, Eastern Russia, and Scotland—and found evidence of the variant only in Nunavut, Canada, suggesting the polymorphism is novel and recently evolved.

## Introduction

The discovery and documentation of structural variants (SVs) has long been important in understanding genomes and genome evolution. For example, chromosomal inversions (CIs) were first described in *Drosophila* early in the 20th century and continue to be described to this day (Sturtevant 1913; reviewed in Wellenreuther and Bernatchez 2018). For the most part, SV documentation in non-model organisms has been limited to CIs because of their large size (spanning several hundred KBs to multiple MBs) making them easier to discover and more likely to influence phenotypic adaptation. Therefore, our knowledge of other sorts of SVs such as copy number variants, chromosomal translocations, and large scale duplications and deletions has lagged behind that of CIs (but see Reid *et al.* 2016; Tigano *et al.* 2018; Wellband *et al.* 2019; reviewed in Merot *et al.* 2020). Interest in SVs—and CIs in particular—stems from homokaryotypes acting like “supergenes” allowing linked alleles to be inherited as a single unit via suppression of recombination in heterokaryotypes (Kirkpatrick 2010; Schwander *et al.* 2014). This allows for the two homokaryotypes to become adapted to different environments and can be a major source of genetic variation linked to phenotypic change (*e.g.*, social behavior, life history development, and alternative reproductive strategies, Thomas *et al.* 2008; Miller *et al.* 2012; Küpper *et al.* 2016; Lamichhaney *et al*. 2016). As a key aim of evolutionary biology is understanding adaptation, the genomics era has resulted in genome-wide data being generated from many non-model organisms allowing the discovery of novel CIs to occur at a rate not previously seen. 

Salmonids are well-studied from a genomic perspective with nine species having at least a draft version of the complete genome (as of January 30, 2021). Recent studies have found SVs in salmonids linked to phenotypic change. For example, rainbow trout (*Oncorhynchus mykiss*) have two CI polymorphisms, the most well documented is a double inversion on chromosome 5 (Omy05). This inversion spans 55 MB and contains more than 1200 protein-coding genes (Pearse *et al.* 2019). Phenotypes associated with the more recent inverted inversion include adaptation to cold water, rapid embryonic and juvenile growth, and the resident ecotype of rainbow trout (*e.g.*, Miller *et al.* 2012; Hale *et al.* 2014; Pearse *et al.* 2014, 2019). The ancestral, non-inverted form, conversely, is associated with warmer temperatures, slower growth, and anadromous or adfluvial ecotypes (*e.g.*, Leitwein *et al.* 2017; Pearse and Campbell 2018). Another ∼13 MB CI polymorphism on Omy20 has been described; however, the function of this CI is less clear (Pearse *et al.* 2019). Other SVs in salmonids have been documented in chum salmon (*Oncorhynchus* *keta*) where an inversion on the sex chromosome has been found in samples across western Alaska (McKinney *et al.* 2020), and in Atlantic salmon where multiple chromosomal translocations and fusions have occurred in different populations (Lehnert *et al* 2019). In addition, two recent studies utilizing whole-genome sequencing have documented many types of SVs (including CIs) in Atlantic salmon and rainbow trout (Bertolotti *et al.* 2020; Liu *et al.* 2021, respectively).

Given the large amount of genome-wide data that have been collected for salmonids, it is surprising that the number of discovered SVs has been limited. This may be due to the secondary nature of documenting SVs where the genomic data have been collected in order to answer questions regarding population assignment and gene flow. As many population genetic approaches require pruning of loci in high linkage disequilibrium (LD)—and high LD is a key feature of SVs—removing these loci will result in SVs being missed during analysis (Lotterhos 2019). In addition, the majority of these genomic studies have utilized RAD-seq approaches where each locus is around 100 bps in length. This makes it difficult to discover small scale SVs and biases the documentation of SVs to chromosomal fusions/fissions and large CIs. Nonetheless, the large number of genome-wide datasets, together with recent improvements in salmonid genome assemblies, make salmonids an excellent group to document novel SVs and determine their presence in multiple populations that span the species’ range (*e.g.*, Lien *et al.* 2016; Pearse *et al.* 2019; Blumstein *et al.* 2020). Therefore, the main aims of this manuscript are twofold, one to re-analyze previously published RAD-seq datasets in Arctic charr (*Salvelinus alpinus*) to document novel SVs, and two, where possible, to determine the presence of novel SVs across the Arctic charr’s range.

## Methods

Three Arctic charr genomic datasets were download from NCBI’s SRA database: one from samples collected around Cambridge Bay, Nunavut (PRJNA413202), one from Eastern Canada around Newfoundland and Labrador (PRJNA655216), one from Scotland (PRJNA607173), and from Russia (PRJNA607173). Note that the Scottish and Russian samples were split for analyses due to the populations being geographically distinct. RAD-seq data were first quality filtered using Trimmomatic (v0.32; Bolger *et al.* 2014) to clip sequencing adapters and remove base pairs with a quality score <20. Quality filtered reads were then aligned against the *Salvelinus* reference genome (GCF_002910315.1) using BWA-MEM with default settings (Li 2013). Alignments were then converted into sorted BAM files using SAMtools in order to facilitate SNP identification (Li *et al*. 2009). ANGSD v0.922 (Korneliussen *et al.* 2014) was used to find and genotype polymorphic positions using a minimum minor allele frequency of 0.05, a posterior cutoff for calling SNP genotypes of 0.95, a maximum SNP *P*-value of 0.00001, and a minimum quality score of 20. The resulting genotype file was outputted in both PLINK and VCF formats for further analysis.

### Locating outlier regions of the genome

In order to locate candidate SVs, we first conducted local principal component analysis (PCA) using the R package “lostruct” (Li and Ralph 2019). Lostruct constructs PCAs based on a user-defined SNP window and characterizes variation between windows. This window-based approach identifiers regions that might be indicative of variation in the structure of the genome. The genome was divided into nonoverlapping windows of 15 SNPs in size and PCA was applied to each window to reflect local population structure. To measure the similarity of patterns of relatedness between windows, Euclidean distances between matrices were calculated for the first two principal components (PCs) and then mapped using multidimensional scaling (MDS) into four dimensions. Candidate SVs were identified by looking for statistical outliers of MDS values for the first four axes (*i.e.*, MDS1 through MDS4). Outliers were calculated using the *boxplot.stats* function in R. Chromosomal regions with more than four outliers for one MDS were categorized as candidates and were further analyzed. The coordinates of the putative inversions were defined by the start position of the first outlier window to the end position of the last outlier window.

Although we used an outlier approach to find variants other genomic processes, such as selection and associated hitchhiking, can elevate LD and cause regions of the genome to become outliers in MDS analysis. Therefore, we performed additional analyses on candidate SVs from the MDS analysis. One of the effects of SVs, and inversions in particular, are suppression of recombination which causes accumulation of mutations at different rates between the two homokaryotypes. A PCA approach targeting loci within the candidate SV should show different clustering patterns compared to the rest of the genome. With this in mind, we used PLINK v1.9 (Purcell *et al.* 2007; Chang *et al.* 2015) to calculate the first two principal components from loci within candidate SVs and the entire genome. If the PCA based on the candidate variant segregated samples into three groups (non-inverted, heterozygous, and inverted)—and this segregation was not found in the PCA on all loci—then this strongly suggests the presence of an SV *vs* selection and hitchhiking (see Huang *et al.* 2020 for a similar methodology in Prairie sunflowers). We also investigated patterns of heterozygosity in loci within candidate SVs. We reasoned that patterns of heterozygosity should differ between the two homozygous versions of the variant. The ancestral version should have higher heterozygosity than the more recent version due to the decreased time for mutation accumulation. To that end, we calculated the average heterozygosity for each individual by summing the total number of observed heterozygous genotypes within any candidate variant and divided that number by the total number of heterozygous sites within the inversion.

### Estimating LD and network analysis

SVs—and CIs in particular—should have a pronounced effect on estimates of LD due to suppression of recombination in heterozygous individuals. Therefore, we estimated chromosome wide LD in PLINK v 1.9 (Purcell *et al.* 2007; Chang *et al.* 2015) for all populations and chromosomes using the following command (–ld-window-r2 0 –r2 inter–chr). Subsequent pairwise estimates of r^2^ were plotted separately for each chromosome in R using the *ggplot2* package (Wickham 2016). Regions of the genome that suggested extended elevated LD were then investigated using a network analysis approach. This was done to determine if SNPs showing elevated LD were grouped in multiple clusters along the same chromosome. For network analysis, SNP pairs with an *r*^2^ < 0.4 were removed. SNPs that were in LD with fewer than four other SNPs were also excluded in order to remove high LD due to close proximity on the same chromosome. Lastly, LD was also measured between loci within candidate variants for each haplotype (*i.e.*, both homozygous and heterozygous versions). We reasoned that structural variation should cause LD to increase between, but not within, different forms of the variant and so should be lower within homokaryotypes.

### Annotation of candidate inversion regions

All protein-coding genes within any candidate inversion regions were downloaded from the charr genome viewer database (GCF_002910315.1). Sequences were uploaded to Blast2Go (v5) and re-annotated against the refseq database. GO terms associated with refseq hits were downloaded and investigated to determine gene function. Assignment of GO terms to blast hits was done with default parameters.

## Results

**Figure 1:**
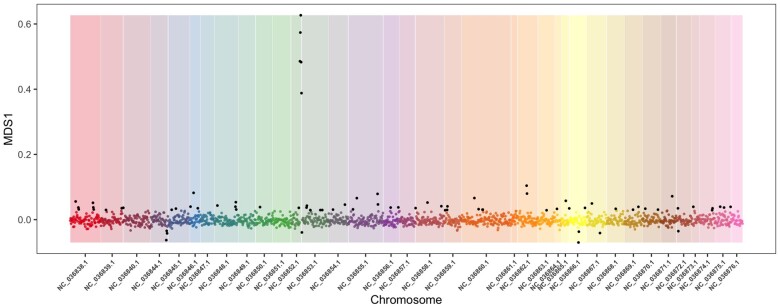
MDS plot of MDS 1 constructed from Arctic charr from Nunavut. Chromosome naming matched that used in Arctic charr genome (Christensen *et al*. 2018). SNP windows classed as outliers (points in black) were determined using a standard boxplot for all data points in R.

**Figure 2:**
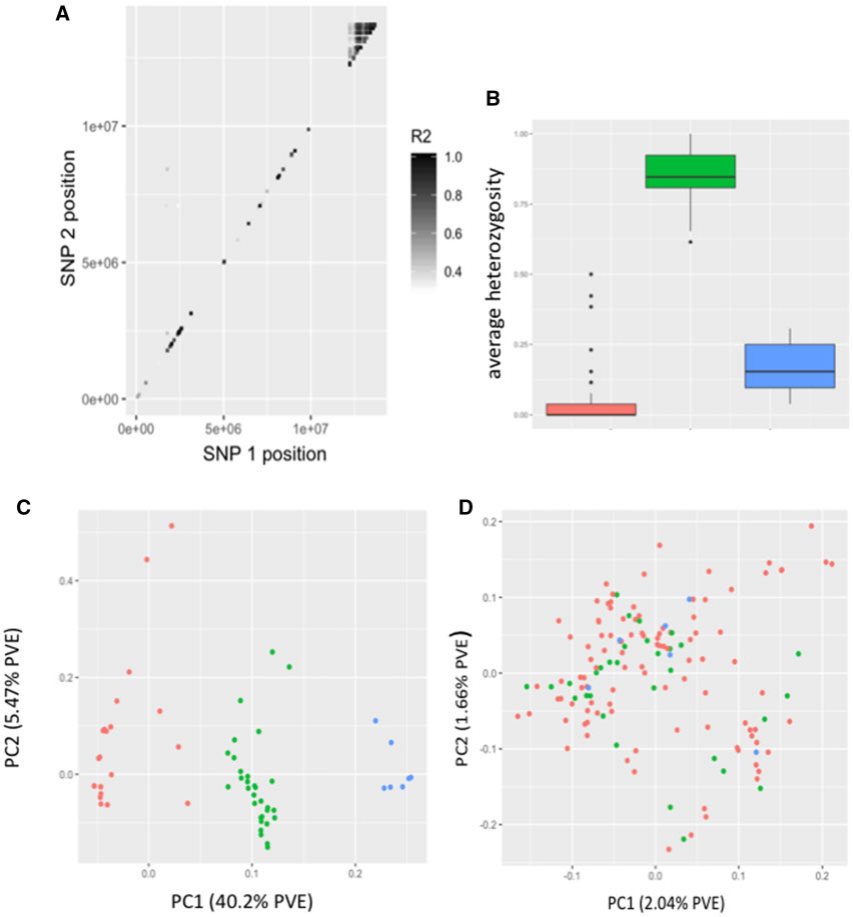
(A) Patterns of LD in Arctic charr from Nunavut for AC12 (NC_036852.1). In order to make the inversion region more obvious, estimates of LD (*r*^2^) were set a minimum value of 0.3. Each point represents a pairwise estimate of LD between the two SNPs. (B) Boxplots showing average heterozygosity for non-inverted, heterozygous, and inverted versions of the AC12 inversion. Boxplots were built from 26 SNPs that are within the two highest MDS1 estimates (see Figure 1). (C) PCA based on SNPs from the 26 loci within the proposed inversion. Three clusters correspond to two homozygote groups (red and blue) and a heterozygous group (green). (D) PCA based on all SNPs from every chromosome. Samples are colored based on their genotype for the candidate inversion in AC12.

Four Arctic charr GBS datasets were investigated (see Table 1). Of which, one dataset—from Nunavut, Canada—showed consistent evidence across all approaches for a candidate SV at the distal end (12.54 Mb to 13.7 Mb) of linkage group 15 (NC_036852.1 BioProject PRJNA413202; Moore *et al.* 2017). This region produced multiple outlier SNPs windows in MDS analysis (see Figure 1), showed elevated estimate of LD (average *r*^2^ = 0.552) compared to the chromosome average (0.02), and patterns of observed heterozygosity clearly showed three groups representing the two versions of the variant, and heterokaryotypes (Figure 2). Alleles at these SNPs showed clear separation between three genotypes with most individuals (72.6%) showing one homozygous version (*i.e.*, the same allele as the *Salvelinus* genome), 23.3% of individuals were heterokaryotypes, and 5% were homozygous for the alternative version (Figure 2B). In addition, a PCA approach showed a lack of clustering when applied to the genome-wide SNP data and the formation of three discrete clusters in the 26 SNPs characteristic of the variant (Figure 2, C and D). Lastly, patterns of LD within homokaryotypes differed compared to analysis of all samples, average *r*^2^ for loci within the candidate variant was 0.067 for the most common form (that matched the *Salvelinus* genome) and 0.147 for the alternative homokaryotype. Based on these results, we refer to this SV below as a candidate chromosome inversion although we acknowledge that other forms of SV could cause similar results.

**Table 1:** Information regarding Arctic charr samples used in the analysis of the candidate inversion

BioProject	Sample area	Number of samples	Number of SNPs	Mean number of SNPs per LG	Number of SNPs in candidate CI
PRJNA413202	Nunavut, Canada	142	19,255	493.7	26
PRJNA655216	Newfoundland/Labrador, Canada	115	31,118	797.8	21
PRJNA607173	Scotland	62	23,005	589.9	12
PRJNA607173	Russia	167	20,156	516.8	10

Note that the total number of SNPs, the average number of SNPs per linkage group, and the number of SNPs within the candidate inversion region were all newly calculated.

### Testing for the presence of the inversion in other populations of Arctic charr

SNPs associated with the candidate inversion were tested in three other populations of charr from the Holarctic region [one population from Eastern Canada (BioProject PRJNA655216), Scotland, and Eastern Russia (BioProject PRJNA607173)]. Each individual possessed the more common homokaryotype genotype (*i.e.*, that which matched the charr genome) as well as frequent recombination between loci within the inversion region. LD patterns did not suggest the presence of the inversion and MDS analysis failed to find any outlier SNP windows in this part of the genome.

### Protein-coding genes within inversion region

A total of 15 protein-coding genes were found in the inversion region. Gene Ontology (GO) terms associated with the protein-coding genes suggested genes involved in ion-binding, cell communication, and ATP binding are present in the inversion region. Interestingly, four genes within the inversion region were annotated with the cellular component term “membrane”. These genes are all located at the beginning of the inversion region [genes interlukin-10 receptor, B and T lymphocyte isoform X1, cholecystokinin receptor, and *FMA155A* (a transmembrane protein)]. See Table 2 for more details on the identity of the genes within the inversion region.

**Table 2:** Functional information for genes within the candidate inversion region

Gene ID	Blast hit	Biological process
XM_023997930.1	Interleukin-10 receptor subunit beta	
XM_023997912.1	B- and T-lymphocyte attenuator	
XM_023997913.1	Cholecystokinin receptor	Gastrin receptor activity
XM_023997850.1	Transmembrane protein FAM155A	
XM_023997853.1	DNA ligase 4	DNA replication, DNA recombination, DNA biosynthesis
XM_023997858.1	Insulin receptor substrate 2	Response to hypoxia, insulin receptor signaling
XM_023997861.1	Cysteine—tRNA ligase	Gonadotrophin-releasing hormone neuronal migration to the hypothalamus
XM_023997868.1	Ankyrin repeat domain	
XM_023997869.1	Pleckstrin homology domain-	
XM_023997872.1	Zinc finger protein 385B	
XM_023997876.1	Integrin alpha-4	Cell adhesion
XM_023997937.1	Neurogenic differentiation factor 1	Endocrine pancreas development; regeneration; negative regulation of Notch signaling pathway; posterior lateral line neuromast hair cell differentiation; inner ear receptor cell development; camera-type eye photoreceptor cell development
XM_023997882.1	Protein phosphatase 1 regulatory subunit 1C	Signal transduction; negative regulation of phosphoprotein phosphatase activity
XM_023997886.1	Nck-associated protein 1 isoform X1	
XM_023997920.1	Zinc finger protein 804A	

GO terms were obtained using NCBI’s RefSeq database.

## Discussion

Interest in documenting SVs stems from the link between genomic variation and the development of phenotypic or ecological adaptation. Most described SVs that have been linked to adaptation concerns CIs due to their large size (frequently MBs in length) and the roles CIs play in reduced recombination between homokaryotypes. Here we document a novel CI that spans ∼1.2 MB on Arctic charr linkage group 12. Given the size of the variant and patterns of heterozygosity and LD (discussed below) we think it likely that this likely represents a CI rather than an alternative form of SV. The inverted version appears to be restricted to charr from the Canadian Arctic in Nunavut, and although the inverted version is rare, our discovery adds to a growing body of evidence that SVs, such as CIs, occur in salmonids and suggests that further SVs are awaiting discovery.

Annotation of the 15 protein-coding genes within the candidate inversion region suggest wide-ranging molecular functions that include protein-binding, cell signaling, and response to hypoxia. The relatively low number of protein-coding genes, makes it unwise to make predictions about the evolutionary forces maintaining the inversion in charr from Nunavut. These samples were taken from high latitudinal environments that are subjected to annual freezing during the northern winter. It is therefore possible that genes involved in oxygen transfer and metabolism may be subjected to strong selection to ensure optimal function. However, only one gene (insulin receptor substrate 2) was annotated with a GO term involved in these processes (response to hypoxia) suggesting alternative mechanisms are responsible for adaptation to high latitude environments and that the molecules involved with these processes are likely not connected to the candidate inversion region.

What is notable about this candidate inversion is its small size. In contrast, most recently discovered inversions appear to be large and contain many protein-coding genes (Wellenreuther and Bernatchez 2018). For example, a CI on the Z chromosome of white-throated sparrows, measure more than 100 MB and contain thousands of protein-coding genes (Tuttle *et al.* 2016). Clearly, large inversions are favored by selection as alleles within many protein-coding genes can be blocked from the effects of recombination. However, we believe there is ascertainment bias in locating large inversions for three reasons: (1) large inversions are more likely to show elevated LD when compared with the rest of the genome, (2) such regions are more likely to be associated with phenotypic variation due to the large number of protein-coding genes located within them, and (3) molecular techniques used for locating polymorphisms in non-model species tend to use a genome reduction approach, such as RAD-seq. We believe this last point is especially pertinent in species with large effective population sizes and/or high recombination rates, as techniques like RAD-seq will locate polymorphic SNPs in a low number of haplotype blocks causing smaller CIs to be missed (*e.g.*, McKinney *et al.* 2016). Considering the frequent use of salmon and trout in population genetic studies it is surprising that, to the best of our knowledge, only three large inversions (*i.e.*, more than 1.5 MB) have been previously reported. However, we feel that this low number is due to the predominance of reduced representation methods (such as RAD-seq) in salmonid genetic studies rather than the rarity of CIs *per se*. The most well-documented salmonid inversion is a ∼55 MB double-inversion on chromosome 5 in rainbow trout. This inversion has been associated with multiple phenotypes including cold-water adaptation, development rate, and life history variation (Sundin *et al* 2005; Nichols *et al.* 2007; Miller *et al*. 2012; Hale *et al.* 2014; Pearse *et al.* 2014; Arostegui *et al.* 2019; Pearse *et al.* 2019). The other two inversions—one ∼14 MB located in chromosome 20 in rainbow trout, and the other ∼20 MB located on linkage group 15 in chum salmon—have not been so intensively studied and no known phenotype appears to be associated with the inversion on chromosome 20 in rainbow trout although the inverted version does appear to more frequent in above barrier resident populations of trout. The inversion on linkage group 15 in chum salmon is associated with the sex-determining region and gives rise to five clusters; XX, X_INV_X, X_INV_X_INV_, XY, and X_INV_Y. Although McKinney *et al.* (2020) were not able to link the inversion to phenotypic effects, the inverted region does contain the gene *Greb1L* which has been linked to run timing in both rainbow trout and Chinook salmon (*e.g.*, Hess *et al.* 2016; Prince *et al* 2017; Micheletti *et al.* 2018). In addition, two recent whole-genome sequencing projects in Atlantic salmon and rainbow trout have documented 242 and six small to medium-sized CIs, respectively (*i.e.*, <1.5 MB; Bertolotti *et al.* 2020; Liu *et al.* 2021). Although the function of these inversions has not yet been determined, many of the CIs reported in Atlantic salmon are known to span multiple protein-coding genes and therefore are likely to influence traits subjected to selection (Bertolotti *et al.* 2020). These two studies suggest that not only are more salmonid SVs awaiting discovery but also that whole-genome sequencing is a more thorough approach to SV discovery than RAD-seq.

When documenting novel SVs, it is important to exclude other processes such as selection sweeps that can cause outliers when using MDS analysis (Li and Ralph 2019) and can show elevated levels of LD compared to the rest of the genome. Such a scenario is especially likely when selection is strong and recombination low which would lead to extensive hitchhiking that could, theoretically, persist for many generations and could cause misidentification of such regions as CIs. Although the candidate inversion reported herein is relatively small (by CI standards), we predict it is unlikely that selection would (1) be specific to this region of the genome—in other words, we failed to find another part of the genome that showed similar outliers in MDS analysis and elevated LD, (2) persist for over 1 MB, (3) cause discrete clustering of three haplotypes when alleles within the CI are examined that are not mirrored genome wide, and (4) cause elevated LD between but not within haplotypes. Furthermore, hard selective sweeps are predicted to generate a continuous pattern of population structure for the candidate region as although hitchhiking can cause linked loci to be in high LD the effect of hitchhiking should decrease even in areas of low recombination as one moves away from the target(s) of selection. Figure 2C clearly shows sharp boundaries to the candidate inversion suggesting some sort of SV has occurred. Moreover, because recombination rate in salmonids is known to increase toward the telomeres (Lien *et al.* 2016), the location of the candidate inversion also suggests a structural polymorphism rather than selection and associated hitchhiking.

Although population genomic approaches can certainly be used to locate candidate inversion, there are limitations to our approaches. Most notably, the need for accurate delimitation of the inversion via long-sequencing of all three haplotypes. This would allow for the breakpoints of the CI to be located and help confirm that the observed patterns documented are due to a CI and not an alternative SV, or selection and recombination. Another limitation is the limited genomic coverage offered by RAD sequencing and other reduced genome sequencing methods. Although such methods will not give false positives, we do believe that RAD-seq will bias detection of inversions to those greater than several MB in size. However, with the continued decrease in sequencing costs, it is likely that more studies will employ low-coverage whole-genome sequencing approaches which should lead to an increase in detection of CIs and other SVs (Bertolotti *et al.* 2020; Liu *et al.* 2021). Indeed, a draft genome of the Atlantic silverside (*Menidia menidia*) describes the presence of 29 inversions including 5 greater than 5 MB (Tigano *et al.* 2021). By using a combination of population structure outliers and elevated patterns of LD techniques we document the presence of a novel CI in a population of Arctic charr in the Canadian Arctic. Our work here demonstrates the capacity for local ordination approaches to detect small candidate inversions and indicates potential presence of additional small SVs detectable within salmonid genomes.

## Data Availability

All raw data analyzed for this manuscript have been uploaded to NCBI’s SRA database and are available using the following accession numbers: PRJNA413202, PRJNA655216, and PRJNA607173.
